# Selecting Privacy-Enhancing Technologies for Managing Health Data Use

**DOI:** 10.3389/fpubh.2022.814163

**Published:** 2022-03-16

**Authors:** Sara Jordan, Clara Fontaine, Rachele Hendricks-Sturrup

**Affiliations:** ^1^Future of Privacy Forum, Washington, DC, United States; ^2^Centre for Quantum Technologies at the National University of Singapore, Singapore, Singapore; ^3^Duke-Margolis Center for Health Policy, Washington, DC, United States

**Keywords:** privacy, health data, genomic, electronic health records, machine learning, privacy engineering

## Abstract

Privacy protection for health data is more than simply stripping datasets of specific identifiers. Privacy protection increasingly means the application of privacy-enhancing technologies (PETs), also known as privacy engineering. Demands for the application of PETs are not yet met with ease of use or even understanding. This paper provides a scope of the current peer-reviewed evidence regarding the practical use or adoption of various PETs for managing health data privacy. We describe the state of knowledge of PETS for the use and exchange of health data specifically and build a practical perspective on the steps needed to improve the standardization of the application of PETs for diverse uses of health data.

## Highlights

- Expert determination for health data de-identification requires understanding current approaches in privacy engineering, specifically the forms and risks or benefits of specific privacy enhancing technologies.
- Expert research on application of privacy enhancing technologies (PETs) to health data is unevenly distributed with respect to applicability to types of health data, actionable guidance on the privacy-data utility tradeoff, and evidence for application to real-world health contexts, including public health.
- Best practices are evolving, but a combination of PETs is currently believed to be most privacy protective.

## Main

Real-world evidence drawn from health data resources is arguably one of the most valuable types of data. The use of real-world data, also referred to as real-world evidence or health “big data,” facilitates research and product development within clinical healthcare and community public health (1–3). Health data is also the most fraught. It is highly sensitive, revealing facts about an individual's health conditions, facts about a person's family members, health practitioners' judgments about a person's psycho-social and health history. With the COVID-19 pandemic, individual health data and public health data can even describe the place of an individual within a state's description of persons permitted or not permitted to access certain amenities (4–6).

Generally, acceptable and lawful uses of real-world health data require that the health data be treated according to the highest privacy standards. In some settings, those lawful uses are more apparent than in others. In most cases, four characteristics determine the sensitivity of real-world evidence:

1. The data is sufficiently granular to identify a specific individual or reveal a particular sample of a population;
2. The level of privacy and security features designed into data collection devices or data transfer systems;
3. The choices that subjects are given to protect the privacy of their data within a single data collection point or multiple and interoperable systems; and
4. The stringency of the governance—law, regulation, policy—of the privacy and security standards for the collection, sharing, and use of real-world evidence.

In the United States specifically, which is the setting for our present analysis, this means abiding by the privacy and de-identification requirements of the Health Insurance Portability and Accountability Act (HIPAA) (7–11). But, HIPAA is mainly silent on precisely what constitutes the highest levels of privacy protections: outside of removing the 18 HIPAA identifiers, other privacy protections are to be decided by an expert. Under the HIPAA expert determination de-identification pathway, it is stated that a “person with appropriate knowledge and experience with generally accepted statistical and scientific principles and methods for rendering information not individually identifiable” (7) can adequately determine that data is de-identified to the point that there is a minimal privacy risk associated with the use of that data (12). Within the context of justifying such determinations, statistical and scientific experts should rely on their knowledge to apply any of several technical methods to achieve a balance between “disclosure risk and data utility.” This includes knowledge of the various privacy engineering techniques and their applicability to health data types and their uses, such as minimizing re-identification risks when data is shared or used to build predictive models (13).

How many PETs should an expert know of? What is the scope of their application? Is there reliable evidence to judge the reliability of the available PETS in a real-world health data use case? To answer these questions, we provide a practitioner's perspective on the relevant research addressing the applicability of seven forms of PETs to minimize re-identification risks in health data use. In this review we intend to share knowledge beyond the simplistic assertions, often seen in commercial literature, that suggests health data protection experts simply transfer differential privacy techniques from protection of [US] Census data for researchers' use to use cases involving electronic health records data. Instead, we suggest that PETs must be tuned to the data environment, including the relevant ethical constraints of the clinical setting, such as fairness, accuracy, and precision of data use.

## PETs Defined and Explained

Wide and varying definitions of PETs persist in the research and practice of privacy engineering due, in part, to the multidisciplinary nature of the field. Privacy engineering, which also encompasses “privacy by architecture,” “privacy by policy,” and “privacy by interaction (14),” brings techniques and theories in from computer science, public policy, cybersecurity, and science and technology studies at least. For example, Wang defines Privacy Enhancing Technologies (PETs) as, “a wide array of technical means for protecting users' privacy (15).” They describe PETs as encompassing everything from privacy policy languages to algorithmic forms of privacy protection, which unify “privacy-engineering methods,” “privacy-engineering techniques,” and “privacy-engineering tools,” and “privacy-by-design (16).”

The wide array of definitions, terminology, and actors make it difficult to identify sources for privacy engineering benchmarking and expertise. Importantly, as we uncovered, the proliferation of disciplines means that there are agreed upon metrics for reporting privacy gains or re-identification risk protections from application of one PET vs. another. Likewise, there are no benchmark datasets against which privacy engineering test can be applied to establish a state of the art. Even when different PETs are applied to machine learning benchmark datasets, such as CIFAR-10 or MIMIC-III, the degree to which they protect privacy while improving algorithmic performance (e.g., ROC-AUC curves) is not described consistently enough to permit rigorous comparison (17–21). Finally, there are few review resources that describe, in comparable language, the applicability of specific PETs to particular forms of health data (22–27).

To get beyond the state of confusion, we accept the broad definition of PETs but arrange them into a framework of three general categories: algorithmic, architectural, or augmentation based. Other efforts to categorize PETs focus primarily on the intersection between hardware types (e.g., mobile devices) and applicable PETs for data types, mostly outside of the health data space (e.g., social media advertisements) (28–30).

Algorithmic PETs include:

1. Homomorphic encryption
2. Differential privacy
3. Zero-knowledge proofs

Architectural PETs include:

1. Federated learning
2. Multi-party computation

Augmentation PETs include:

1. Synthetic data
2. Digital twinning

Algorithmic PETs protect privacy by altering how data is represented (e.g., encryption, summary statistics) while still containing the necessary information to enable use. They provide mathematical rigor and measurability to privacy. Unlike algorithmic PETs, Architectural PETs are grounded in the structure of data or computation environments. The focus is on confidential exchange of information among multiple parties without sharing the underlying data. Augmentation PETs protect privacy by using historical distributions to direct generation of realistic data that augments existing data sources. Augmentation PETs can be used to enhance small datasets or to generate fully synthetic datasets, including multiple interrelated datasets constitutive of a simulated system, which can augment the overall range of useful and available datasets. Although we arrange the landscape of PETs into different categories, it is important to note that they are often used in combination to fill privacy, security, or data sovereignty needs as the use case requires.

## State of the Peer-Reviewed Literature

If experts must make a determination regarding the privacy techniques necessary for use of real-world health data, where should they turn for guidance? Apart from direct consultation with other experts, examination of the peer-reviewed literature on the topic would be defensible sources of guidance. As domain experts in health data privacy, machine learning, and computer science, we are concerned that there is inconsistency, opacity, or outright absence of clear discussions about the usefulness of PETs for health data use in the peer-reviewed literature. In our examination of the state of peer-reviewed literature below, we focused on the intersections between PETs and health data applications, in three domain expert repositories: ACM Full-Text Collection, IEEE Xplore, and PubMed. From the list of retrieved articles, we selected primary-research papers that described the development or use of a PET(s) for health data applications, then categorized the articles based on the primary PET they investigated. We went a step further to evaluate the usefulness of the results for a practitioners' audience, as described in the next section of the paper.

Table 1, ”A Survey of the Peer-Reviewed Literature on Use of PETs for Healthcare Data” below shows the variation of search terms used for each PET and health data application, as well as the number of articles retrieved from each database.

**Table 1:** A survey of the peer-reviewed literature on use of PETs for healthcare data.

**Keywords/Search strategy**	**Privacy-preserving computational method: number of Articles retrieved per database per search query**					
	***“zero-knowledge proof”***	***“homomorphic encryption”***	***(“federated learning” OR “federated deep learning” OR “federated machine learning”)***	***(“secure multiparty computation” OR “secure computation” OR “multi-party computation”)***	***“differential privacy”***	***“synthetic data”***
AND (”electronic health record“ OR ”electronic medical record“ OR ”EHR“ OR ”EMR“ OR “PHR”)	IEEE: 0 ACM: 0 PubMed: 0	IEEE: 2 ACM: 33 PubMed: 3	IEEE: 6 ACM: 8 PubMed: 9	IEEE: 27 ACM: 30 PubMed: 1	IEEE: 7 ACM: 80 PubMed: 5	IEE: 27 ACM: 101 PubMed: 17
AND (”direct to consumer genetic testing“ OR ”consumer genetic testing“ OR ”ancestry testing“ OR ”genetic testing“ OR ”personalized medicine“)	IEEE: 0 ACM: 0 PubMed: 0	IEEE: 4 ACM: 7 PubMed: 4	IEEE: 0 ACM: 2 PubMed: 3	IEEE: 4 ACM: 25 PubMed: 2	IEEE: 1 ACM: 31 PubMed: 0	IEEE: 9 ACM: 21 PubMed: 4
AND (“medical” OR “health”) AND (”direct to consumer artificial intelligence“ OR ”consumer artificial intelligence“ OR “artificial intelligence” OR “machine learning”)	IEEE: 0 ACM: 0 PubMed: 0	IEEE: 2 ACM: 16 PubMed: 3	IEEE: 3 ACM: 7 PubMed: 17	IEEE: 4 ACM: 22 PubMed: 2	IEEE: 3 ACM: 36 PubMed: 4	IEEE: 87 ACM: 153 PubMed: 40
AND (”medicine“ OR ”medical“ OR ”health“) AND (”mobile app“ OR ”mobile application“ OR “mobile”)	IEEE: 5 ACM: 0 PubMed: 0	IEEE: 1 ACM: 27 PubMed: 1	IEEE: 28 ACM: 82 PubMed: 4	IEEE: 3 ACM: 122 PubMed: 1	IEEE: 15 ACM: 274 PubMed: 4	IEEE: 11 ACM: 271 PubMed: 6
AND (”medicine“ OR ”health“ OR “medical”) AND (”x-ray“ OR ”imaging“ OR ”CT“ OR ”MRI“ OR ”PET")	IEEE: 0 ACM: 0	IEEE: 8 ACM: 197 PubMed: 1	IEEE: 0 ACM: 11 PubMed: 72	IEEE: 58 ACM: 56 PubMed: 0	IEEE: 0 ACM: 89 PubMed: 1	IEEE: 0 ACM: 249 PubMed: 145

## Challenges for Expert Determination of Usefulness of Peer-Reviewed Literature on PETs and Health data

We adopted a two-step approach to classifying of articles with practical utility for individuals working with health data. First, we ranked each article for its applicability to the health data context and then weighted each article according to the level of testing rigor described in the article. Applicability to the health data context was demonstrated by description of application of any of the PETs examined here to specific types of health data, such as Genome Wide Association Studies (GWAS) or electronic health records (EHRs). The level of rigor for testing of the PET was measured on a three-point scale based upon whether the article reported only theoretical application of a specific PET, used a single source of health data or strictly benchmark datasets from other fields, or whether the article described application of the PET to analyses using multiple datasets including benchmark and health-specific data. We also initially evaluated the articles according to the detail given about the privacy-utility tradeoffs and the computational time or hardware required to use the PET, but found that this was a rare feature in the literature we found.

Application of our two-step method for evaluation of the literature revealed a weakness in applicability of the recommendation, common among privacy engineers and machine learning experts, that domain expertise should guide privacy decision-making, such as determining appropriate thresholds for privacy performance (33). We found that our small team of three researchers, whose academic and professional background includes engineering, privacy engineering, computer science, machine learning, health data de-identification and health data privacy, evaluated the PET literature differently, even when working with a similar rubric and consulting one another often. We found that our individual determinations of the relevance of research reviewed was dependent on knowledge of health conditions, recognition of the names of benchmark datasets (e.g., the Wisconsin Breast Cancer dataset), or ability to interpret machine learning performance metrics, such as such as ROC-AUC curves, in this context (34). Evaluation of the rigor of testing was also uneven between us as articles often failed to elaborate clearly on the meaning of performance metrics for privacy preservation generally, for privacy protection of health data, or for resolving the practical task of determining a privacy parameter in the face of practical data analysis. Whether measured as similarity of scores for healthcare relevance or for rigor of testing, our team achieved only 66% similarity on over 80% of the resources reviewed. Our experience raises specific questions regarding the application of the “expert determination” clause for HIPAA, namely “what makes a relevant expert?,” and also echoes the existing general questions about the limits to expert collaboration in engineering and computation task domains (35).

## Algorithmic PETS

### Differential Privacy

Differential privacy (DP), which is a set of rigorous mathematical changes made to mask sensitive data points is known widely due to its application to the US Census 2020 data. DP is one of the few PETs with known methods and measures to define and guarantee privacy (36, 37). In the case of DP, privacy is defined as an adversary's inability to tell whether an individual is part of a dataset or not, even if the adversary has complete knowledge of the individual's data, as well as over all the other entities in the dataset. Differential privacy ensures privacy by making individuals' data obscure so that the inclusion or exclusion of one individual's record makes no statistical difference on the output. Data is made differentially private by adding noise, through either mathematical transformations of data points (e.g., Laplacian, neighbor distance) or modification of data through algorithmic manipulations (e.g., genetic algorithms). Selection of the transformations or algorithmic changes must be compatible with the type of data ranging from genome-wide association studies (GWAS) to physical sensor data.

DEFINITION 1. (Differential Privacy (6)) A randomized function A gives ϵ-differential privacy if for all datasets D and D′ differing at most one row, and all S ⊆ Range (A) Pr [A(D) ∈ S] ≤ e ϵ Pr[A (D′) ∈ S]

DP is a demanding privacy standard, even when epsilon, the constraining mathematical constant, is large (>1). To address the challenges of meeting DP while maintaining data utility, researchers have modified epsilon values and other DP constraints to create new measurable privacy standards, such as crowd-blending privacy, coupled-worlds privacy, membership privacy, and differentiability. However, these modifications of the strong assumptions of DP create weaker protections against adversaries, and thus introduce greater risks of privacy loss. Differential privacy is mathematically rigorous and relatively well known, but the application of DP presents problems for health data use. The data use problems of DP include the inapplicability of DP to time-series data and that DP may homogenize outliers in a dataset, thus reducing the diversity in the dataset (17). Diversity problems can raise unique problems for applicability of DP in healthcare as outliers' values are often clinically and financially meaningful. Further, use of DP is not well suited to cases where firms are seeking an off-the-shelf privacy solution for use in health care, for example where non-linear models are used or where the effect of DP “washing out” group influence is detrimental to answering questions about a diverse client population.

While many of the articles reviewed here were explicit about the tradeoffs in utility, privacy, and computational time at specific values of epsilon (e.g., 1.0, 0.01, 0.001) and with weaker variations of DP, the reporting about what types or granularity of private information could be revealed at each level was incomparable. This made it difficult to draw an expert judgment about whether a specific level or form of DP is acceptable for the privacy and utility needs of the health data. When blended with other PETs, DP has promise for the healthcare setting. But the cost of combining these approaches, whether measured as loss of data utility or increase in computational resources, is not well described in the research literature. As a result, experts are unable to reliably use this research to substantiate an expert determination that DP should be used.

### Homomorphic Encryption

Health data privacy demands can be met through excellent cybersecurity and data encryption practices. However, securing data through encryption could cause complications for organizations that want to perform analyses on their encrypted data. They are faced with the dilemma that decrypting data opens that data to the risk of discovery but analysis of data while encrypted is severely limited. Homomorphic encryption is a highly secure, strongly privacy-preserving method that allows computational analysis over encrypted data. But those operations are limited to multiplication or addition of encrypted values.

A scheme is additively homomorphic if [x] ⊕ [y] = [x + y] and multiplicatively homomorphic if [x] ⊗ [y] = [x · y] where [*x or y*] denotes the encrypted or ciphertext value of plaintext *x or y*, ⊕ denotes homomorphic addition, and ⊗ denotes homomorphic multiplication. Put simply, encryption after computation is equivalent to computation after encryption.

Homomorphic encryption schemes can be partially, somewhat, or fully homomorphic. Fully homomorphic encryption (FHE) schemes are the most computationally complex, as they can perform arbitrary addition and multiplication over encrypted data. They are often based on strongly non-linear functions and modular arithmetic over large numbers. Partially homomorphic encryption (PHE) schemes perform one operation (e.g., addition or multiplication) an arbitrary number of times over encrypted data. Somewhat homomorphic encryption (SHE) schemes perform specific numbers of rounds of computation using one operation over encrypted data or allows for a mixture of operations on limited data.

Homomorphic encryption is first and foremost used to provide strong security to sensitive data while enabling private computation. There are two core security features:

1. One-wayness: No efficient adversary has any significant chance of linking the corresponding plaintext to the ciphertext when they only see the ciphertext and the public key of the patient held by the data owner.
2. Semantic security: An adversary will be unable to distinguish whether a given ciphertext is the encrypted version of two different plaintexts.

Here, plaintext refers to unencrypted data, and ciphertext refers to encrypted data.

How has HE been used in health data research? One way is within third-party computation services where an objective is to maintain the highest level of data privacy, harden the service provider's algorithm or model against discovery, and restrict access to the computation results.

For example, Vizitiu implemented a modified FHE scheme called MORE (Matrix Operation for Randomization or Encryption) based on linear transformations to support third-party deep learning (38), that demonstrated that neural networks be trained a wide range of homomorphically encrypted data types including whole-body circulation multivariate data, X-ray coronary angiography images, and the MNIST handwritten digits images. Those networks can use virtually any algorithmic type, such as regression and multiclass classification problems. The client's sensitive medical data stays private during training, the client's result stays private during inference, and the neural network parameters remain confidential to the model owner. When comparing this privacy-preserving scheme with a neural network trained and operating on unencrypted data, the computational results were found to be indistinguishable, and runtimes increase only marginally.

HE can also support secure data storage and processing in which patient data is kept private from medical units, medical units can only access parts of patient data for which it is authorized, and the medical units' tests and analyses are kept private from patients. For example, Ayday et al. implemented privacy-preserving disease susceptibility test (PDS) that stores patients' encrypted genomic data and pseudonymized identifiers at a storage and processing unit (39). Medical units can retrieve encrypted genomic data to which they are authorized and compute their susceptibility tests using homomorphic operations such as weighted averaging on encrypted SNPs. However, and importantly for insider threat mitigation, a curious party at the storage processing unit would be unable to infer the contents of the patient's DNA from their stored data. They evaluated the system using a real human DNA profile (40) and markers for real diseases (41, 42). They found that the encryption, homomorphic operations, and decryption computation times and the memory requirements did not diminish the practicality of the algorithm, with all operations being several orders of magnitude faster than the sequencing and analysis of a sequence.

Over the past few years, researchers have proven that HE can rigorously meet security requirements for sensitive medical data. However, the high security benefits come with a dramatic tradeoff in performance, as encrypted computations are often several orders of magnitude slower than plaintext computations and schemes require high levels of memory. This limits the PET's practicality in the real world, especially in resource-constrained environments such as mobile or IoT devices (43). We may overcome this barrier by using simpler or modified HE strategies, but at the cost of weaker security. We recommend that this performance-privacy tradeoff be considered carefully before deciding which HE scheme to implement, and that the research field explore more diverse variations of HE and examine their performance-privacy tradeoff in diverse application spaces.

### Zero-Knowledge Proofs

One of the reasons why health data is de-identified in the first place is for it to be securely transferred between organizations with a legitimate use for the data. A challenge for health data privacy engineers is determining who is a legitimate user. While there are stipulations in the HIPAA regulations that address data access for “business associates” and so forth, verifying the digital identity of data receivers is a technical challenge not covered under the applicable regulations. One of the PETs discussed in privacy engineers—zero-knowledge proofs (ZKP)—represent a method for verification of sensitive healthcare data between collaborators without explicitly transferring the data. ZKP may be particularly for medical Internet of Things (IoT) applications (44). Alas, ZKPs are the least well characterized and most infrequently discussed in research papers addressing health data privacy.

ZKP is a method for ensuring security and privacy of data that is borrowed from cryptography. According to (43), “A zero-knowledge proof enables the prover (P) to make sure the verifier (V) is certain that some statements are correct, but the verifier (V) does not learn anything except the validity of the statement.” ZKP theoretically allows sharing of data securely and privately across multiple devices but with potentially high computational and communications costs.

The most fruitful application of ZKPs for healthcare data will likely emerge in the areas of identity and attribute verification (44–46). Creating secure communications across medical devices will become more relevant with the increase in diversity and span of medical sensor networks, including body area networks (47). Overcoming the architectural challenges posed by the medical internet of things, such as device diversity and communications latencies and the variability of computational resources available to validate proofs is a key challenge to moving ZKP from theoretical to practical application (46).

## Architectural PETs

### Federated Learning

Following the maxim that personal information privacy is best maintained when individuals control their own information, privacy engineers turned to distributed computing to circumnavigate the challenge of protecting users' data by putting data protection into the hands of local data owners. Federated Learning (FL) localizes the control of data and even the control of models running on that data. To the extent that data owners prefer strict sovereignty over their data and prefer models that are trained with the most diverse data, even where that data is not strictly available to them, then FL is a useful PET to employ.

**Figure 1:**
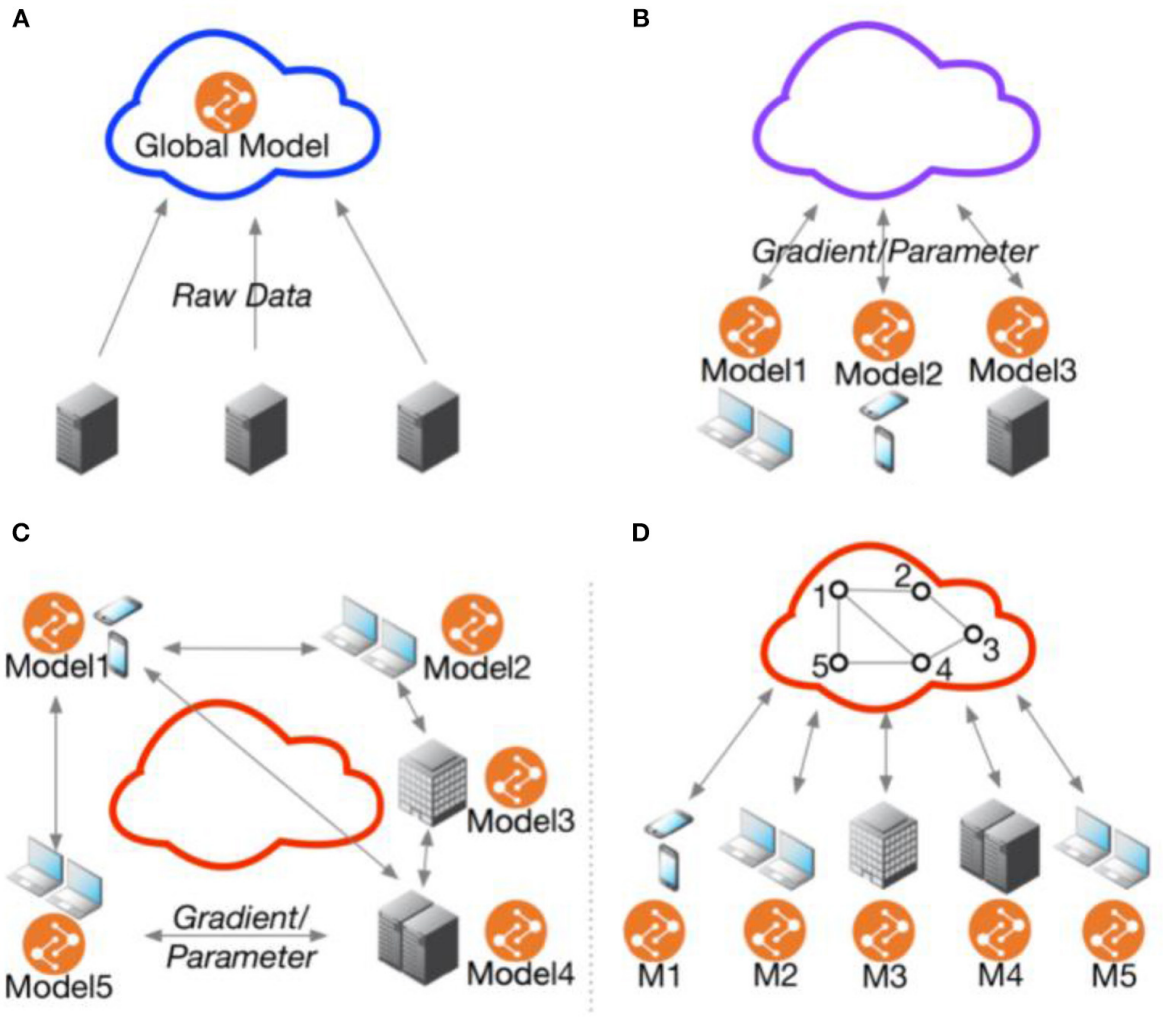
Federated multi-task learning topology. **(A)** Cloud-based distributed learning; **(B)** Centralized ederated learning; **(C)** Decentralized federated learning; **(D)** Centralized communication topology with decentralized parameter exchanging topology. Adopted from He et al. (48).

Federated Learning (FL)is an architectural PET that confers a few advantages over algorithmic PETs (see Table 2, Figure 1). These advantages are primarily data use related: first, FL is data agnostic; there are no a priori restrictions on the types of data that can be made more private through this PET. Second, FL is (theoretically at least) algorithmically agnostic: it can be used as a data architecture in the background of any form of machine learning and the various algorithmic PETs described elsewhere in this resource can be applied with FL. Third, in a decentralized FL system, an honest-but-curious adversary or an adversary intent on data exfiltration has limited access to the data present in one node. This reduces the size of the privacy loss from data breach. Fourth, FL can improve model performance by giving access to a greater diversity of training data, provided that the challenges of interoperability are addressed as well.

**Table 2:** Typology of federated learning.

**Typology of federated learning** **(****31****)**		
	**Data**	**Models**
Decentralized	Data remains on users' devices or on facility servers and models are sent to those devices or servers for training. Weights are sent back to the model server in raw or aggregate form. This may also be called cross-silo federated learning.	Model components are partitioned and sent to a sample of devices, which train model partitions on device and returns weights to the model server directly or *via* an intermediate aggregation server. (32)
Centralized	Data is centralized then partitioned for sharing out to others to boost their data and local model training. This may also be called data center distributed learning.	Data from decentralized devices is ingested into a central location for model training and new models are sent back to disaggregated locations after training on the centralized data and servers. (20)

FL invites some disadvantages because it is an architectural privacy solution. First, it is not clear when a federated approach is the best choice for the specific reason of protecting data privacy. While the architectural approach allows for data sovereignty, this choice decentralizes the protection of data at each node to that node, creating challenges for balancing both privacy and security. This presents a challenge for establishing a true baseline of privacy across the learning system. Second, the decision to apply centralized vs. decentralized FL must be constrained by other side constraints, such as data owners' preference for strict or loose data sovereignty, communications latencies, device and data heterogeneity, and the ability of collaborators to agree on interoperable terms for raw data or features (48). Third, although FL works with virtually any form of data, from electronic health records to medical images, the data collected from disaggregated clients are often coming from different distributions. As a result, the data is not independent and identically distributed (IID), which is a key assumption for many statistical tests and machine learning models. For example, medical institutions in different neighborhoods would likely see patients of different demographics and thus are unlikely to have the same distributions. FL with non-IID data may present the challenge of achieving high model accuracy and generalization in some health data applications (18, 21, 49). Other strategies such as data similarity clustering have demonstrated potential in overcoming this challenge without increased data centralization (50). Additionally, it's important to note that FL with IID data does not always outperform that with non-IID data, and it is unclear what characteristics of health data may result in superior model performance with non-IID data.

A unique feature of Federated Learning (FL) is its utility for privacy-preserving machine learning. Federated learning is used for collaborative training of machine-learning models over a set of training or testing data sources that are kept local to the data owners. Applied in the medical space, it enables patients, medical professionals, and medical institutions to gain insights collaboratively without moving private data, whether records, images, or sensor data, beyond the security parameters and risk appetites of the data owners. As Kairouz et al. describe it,

“One of the primary attractions of the federated learning model is that it can provide a level of privacy to participating users through data minimization: the raw user data never leaves the device, and only updates to models (e.g., gradient updates) are sent to the central server. These model updates are more focused on the learning task at hand than is the raw data (i.e., they contain strictly no additional information about the user, and typically significantly less, compared to the raw data), and the individual updates only need to be held ephemerally by the server. While these features can offer significant practical privacy improvements over centralizing all the training data, there is still no formal guarantee of privacy in this baseline federated learning model (31).”

Distributed computing carries costs, such as latency from transfer. However, there are also benefits: FL models perform consistently better than local models trained on just a single party's data (51). In fact, FL performs comparably to explicit data-sharing, with models trained in an FL architecture being almost perfectly comparable on model quality indicators achieved with centralized data.

While federated learning is gathering attention from health data experts intent to protect privacy, particularly for mobile health applications, there are important differences in the privacy, security, and feasibility costs of implementing different varieties of FL, specifically centralized vs. decentralized federated learning (52). First, it is important to emphasize that centralized and decentralized can mean both adaptations to the topology or structure of a federated learning system *or* to the arrangement of data in that system. For example, centralization in some federated learning systems means that a central server directs the distribution of model training across the multiple devices or institutions collaborating. The central server coordinates training and updates, including the selection of nodes for participation based upon pre-defined eligibility criteria, such as the presence of a trusted network key. One challenge with this approach is that it may be infeasible to have a center that is trusted by all potentially collaborating nodes.

With respect to decentralization, decentralization may mean disaggregation of data, whether because the data is never aggregated to begin with, or it may mean decentralization of model training. Decentralizing the training of models may introduce problems of communication between servers and devices and may also present challenges for maintaining high levels of privacy when data is stored and shared by users. In a peer-to-peer topology, which is a highly decentralized form of FL, communication is between clients and edges on a dynamic network graph. In this network, each node communicates with only a few of the other nodes, potentially limiting the rate of learning due to communications bottlenecks between nodes with different resource constraints. However, there are three general advantages to decentralized federated learning: “(1) data privacy could be preserved better than the centralized case; (2) the computational burden is released by SGD and parallel computing compared with the centralized GD/SGD processing (a linear speedup); (3) the communication efficiency can be increased significantly by adopting FL (32).”

Limitations to federated learning are that the scalability of FL is dependent on collaboration and stable communication between otherwise “sovereign” and asymmetrical devices or institutions. As the scale or diversity of a federated network increases, performance loss may impair the performance of the model learned whether that performance loss is due to communications lags, device heterogeneity, inefficient data partitioning, or poor choice of hyperparameters that restrict model updating and communication rounds. The FL research has not delivered consistent reporting of the number of nodes, diversity of nodes, optimal communication speed and rounds, and whether these parameters affect the privacy gains proposed as the reason for using FL.

### Secure Multiparty Computation

Secure multiparty computation, variously described as SMC or SMPC, is a form of distributed computing that enables computation across multiple encrypted data sources while ensuring no party learns the private data of another. The two fundamental properties of SMC protocols are:

1. Input privacy: no party can make inferences about the private data held by other parties from the messages being sent.
2. Correctness: either the protocol is robust and honest and parties are guaranteed to compute the correct output, or the protocol aborts if they find an error.

Under the umbrella of SMPC, there are two-party and multi-party computation protocols. Protocols include, but are not limited to, garbled circuits, secret sharing, and oblivious transfer. Secret sharing protocols distribute data to collaborators to allow them to perform necessary computations but without granting direct access to the data. Garbled circuit protocols two parties, a garbler and an evaluator, to compute a known function on their jointly held data. To do so, the garbler sends encrypted truth tables to the evaluator which can only be decrypted using a key (token) sent *via* oblivious transfer.

How has SMC been used in health data research? While research into SMC applied directly to healthcare data is still in its early stages, existing literature has demonstrated its ability to support collaborative inference and third-party model training for image and multivariate data.

For the application of collaborative inference, Ismat et al. presented PRICURE, a system to enable privacy-preserving collaborative prediction between multiple parties holding complementary datasets on the same machine learning task. In other words, clients can receive a single aggregated result constructed from several private machine-learning model owners (22). Thanks to additive secret sharing, the system keeps the client's input private from the model owners, and the model parameters private from the clients. Their experiments used image-based and multivariate data—the well known MNIST, Fashion-MNIST, IDC breast cancer, and MIMIC in-ICU length-of-stay datasets—as well as feed-forward neural network models. Their performance evaluation demonstrated a very minimal tradeoff on inference accuracy, tested scalability of up to 50 model owners, and negligible overhead for secret-sharing.

For the application of third-party model training, Wang et al. leveraged PHE and designed seven secure computation protocols to allow a cloud server to efficiently and securely compute simple computations for multiple parties (53). To demonstrate the protocols' utility, they outsource Secure Vector Machine (SVM) training on a heart disease dataset and a diabetic retinopathy Debrecen dataset. In this demonstration, both the data samples and training parameters for the SVM model are kept private from the cloud server responsible for model training.

There have been efforts to leverage SMC in genomic testing and genome-wide association studies meta-analysis (54–56). However, these applications so far are mostly theoretical and seem to simply take a well established non-healthcare SMC solution and apply it directly to genomic data without much consideration to the specific requirements of the healthcare space.

Although SMC offers one of the strongest privacy protections for all the participating parties, the communication and computational complexity of SMC is too high to be used reasonably on computations at scale and in resource-constrained environments such as wearables and mobile devices. PRICURE was shown to be scalable, but only until the computational burden of inference overwhelms the system. Although Wang et al.'s solution is more efficient than previous work, the experimental overhead for training was still too high for real-world deployment. Both articles stressed the importance of using optimization through hardware acceleration, parallelization, and efficiency advances to enable real-world deployment of SMC.

We recommend that more research focus on practical SMC solutions for both resource-constrained environments as well as computations at scale to overcome challenges in performance efficiency. Presently, SMC cannot be easily leveraged in healthcare.

## Augmentation PETs

### Synthetic Data [SD]

Synthesizing data (SD) may be the most effective way to maximize privacy for patients or providers: whether it is fully or partially synthetic, SD does not contain direct, identifiable measurement of actual patients or events. Fully synthetic data involves the synthesis of all records while partially synthetic data entails the synthesis of only sensitive information. With an increasing number of data synthesis packages in commonly used languages like Python and R, data synthesis is becoming easier and more cost-efficient, allowing organizations to build statistically defensible, privacy-preserving, data to support the rapid development of ML on more balanced datasets. Synthetic data can be particularly useful when data needs to be rebalanced to address class imbalances that arise due to a dearth of data from small samples of persons who, if their data were included in available datasets, could be easily re-identified due to the rarity of their condition. It is also useful when efforts to create models for healthcare applications should not be trained on real patient data due to re-identification risks. For example, synthetic data could be used to truly measure the utility loss that arises from the use of algorithmic PETs or to measure the risk for data loss from architectural PETS.

Limitations to the use of synthetic data arise from methods for generating data, types of data that can be synthesized (categorical and discrete vs. image *via* GANS), and the need to validate that the synthetic data is representative of real data (57). Other limitations include findings suggesting that “compared with models trained and tested on real data, almost all machine learning models have a slightly lower accuracy when trained on synthetic data and tested on real data across all synthesizers and for all machine learning models analyzed (34)” Other challenges to the uses of synthetic data are non-realistic occurrences that arise in synthetic data (58) including that synthetic data is simply more refined than “wild type” data (59). Finally, for individuals grappling with the challenge of creating synthetic data for privacy-preserving healthcare applications, there are relatively few resources available in the peer-reviewed literature that describes methods for synthesizing data from messy, multi-source, health data sources. With these limitations in mind, uses of SD should be restricted to secondary uses that require realistic but not real data.

### Digital Twins [DT]

In our review of the privacy enhancing technologies literature, there was an emerging, but limited, discussion of the applications of “digital twins” (DTs) to the protection of data about healthcare institutions and healthcare systems (60). Digital twins are “virtual representations of what has been manufactured” that serve as virtual counterparts to physical entities ranging from persons to hospitals against which new systems or products can be tested or integrated without threat of failure (61). DT combines AI, IoT, and sensor networks into a “digital cocktail” (62) that creates a real-time simulated environment in which various tools can be tested without potentially exposing real persons or facilities to unknown harms. Invoking methods akin to partial data synthesis, digital twins rely on real-time data flows to build virtual models of behavior against which other machine learning models could be tested without opening an attack surface that could expose the patient's or physical plant's true data (63).

At present, the privacy protections of digital twins are not well characterized; whether a digital twin of a patient or patient population effectively protects the physical patient or patients from re-identification from an external adversary who is otherwise unaware of the identity of the physical twin has not been tested. As digital twins unify multiple forms of data and many different systems, characterization of the overall privacy risks of digital twins will turn on key questions already invoked in this Perspective piece. For example, whether there are additive or multiplicative benefits from the joint use of digital twins and another PET, such as differential privacy, creates significant loss of data utility, significant drag on computational time, or significant privacy risks through creation of the system is also unknown. At present, the application of digital twinning as a PET in healthcare should be considered primarily theoretical.

## Expertly Choosing PETs for Health Data

Experts in health data management, public health, computer science, and privacy engineering may face certain challenges in their collaborative attempts to ascertain the state of the literature on PETs and health data. Our present experience lends us to recommend that further collaborative attempts to scope or systematically review the literature on PETs and health data should leverage a broader range of literature search tools and databases (e.g., Google Scholar and Web of Science Scopus) for a potentially greater chance at reaching expert consensus or determination in choosing PETs for health data management and use.

Most resources available to assist data scientists with HIPAA privacy compliance focus on applying the HIPAA “Safe Harbor” method, under which the 18 personal identifiers are removed (7). However, experts on data re-identification have challenged the effectiveness of the Safe Harbor standard to ensure privacy or at least reduce the risk of re-identification to the “very small risk” level expected (64).

Alternatively, the expert determination provision in HIPAA offers opportunities for experts to push for the application of novel techniques, such as any of the PETs described above. Under the HIPAA expert determination pathway, addressing inherent privacy risks while preserving data utility through obscuring patient identifiers is the goal. Therefore, as the expert determination pathway continuously requires experts to rely on their knowledge of state-of the art technical methods to balance disclosure risk and data utility, the following themes from the literature are important to remember:

1. Not all types of health data can be protected with each PET without significant loss of data utility or significantly more computational resources required.
2. While architectural PETs create opportunities for data sovereignty and privacy in the aggregate, these do not protect data privacy in the local environment.
3. The state of the art of research on PETs for health data often uses well worn benchmark datasets from other domains (e.g., CIFAR-10) or specific health data types (e.g., MIMIC-III) that cannot be easily extended to health data in the wild.
4. The combination of architectural and algorithmic approaches is the emerging best practice in the research literature. Which combinations achieve the highest privacy protections with the lowest utility loss are dependent on the data, computational resources, and thresholds for data utility loss.
5. When to use a specific PET is context-dependent—the choice of PET in a pre-inferential setting will vary from the choices to be made in a post-inference setting—and steady use of a single method will result in unintended privacy loss.
6. None of the algorithmic and architectural PETs can guarantee the privacy of individuals represented in the data. Fully synthetic data is the closest to providing such guarantees, but there remains the possibility that fiction may mimic fact, leading to phantom re-identification (65).

## Discussion: PETs in Public Health Practice

Public health entities and their key health system stakeholders should fully consider the application of PETs and privacy engineering overall to better protect health data privacy. Mindful integration of digital technologies into health can help to remove the gaps in the real-world evidence base needed to improve global health. But, this should be done in a way that minimizes harmful disruptions to health infrastructure and re-centers the value of health privacy for people and communities. Integration of novel technologies, like PETs, must re-invigorate the mission of public health and health care entities to ensure privacy throughout the management, use, and sharing of health and health-relevant data during and beyond the COVID-19 pandemic (66). Understanding both the stated benefits and limitations to each PET is mission-critical to these goals.

Our review of the literature leads us to the unfortunate conclusion that there remains much work to be done to make PETs a normal part of everyday expert practice of privacy and security for health data. Instead, we found an uneven collection of research articles from multidisciplinary experts in a burgeoning field who struggle to define health data privacy benchmarks, performance metrics, and common vocabulary. In the absence of better and standardized guidance, an expert determination of which privacy engineering approach to take may be a matter of taste or convenience.

To move the health data privacy community forward on this topic, we provide the above review and also the summary in Table 3. Table 3, “Key Characteristics and Considerations of Each PET” is an overview of each algorithmic, architectural, and data augmentation PET described, specific use cases, pros and cons associated with the use of each PET in health data contexts, and opportunities for future research. We recommend that public health entities collaborate with policymakers and healthcare system stakeholders to deeply explore possibilities of leveraging the expert determination provision in HIPAA and determine which PET method(s) and respective use case(s) best apply.

**Table 3:** Key characteristics and considerations for each pet.

**PET**	**Description**	**Use-cases**	**Pros**	**Cons**	**Future research**
Differential Privacy	Adds noise to a dataset to reduce an adversary's ability to tell whether an individual is part of the dataset Some variations improve data utility at the cost of weaker privacy protection	Publishing or sharing data to satisfy research needs	Provides measurable privacy guarantees	Privacy-utility tradeoff Inapplicable to time-series data Under-represented or “unique” minority data may not be well-characterized	Comparable and consistent reporting between DP variations of types and granularity of at-risk private information
Homomorphic Encryption	Encryption scheme that enables private computation over encrypted sensitive data Partial, somewhat, and fully homomorphic encryption	Third-party computation Data storage and processing	Provides a high level of privacy Compatible with most data types	Inefficient, expensive, and complex Not well-suited to resource-constrained environments	Explore more diverse and lightweight variations of HE especially for resource-constrained environments Analyze performance-privacy tradeoffs carefully
Zero-knowledge proofs	Verification of sensitive data between collaborators without explicitly transferring data	Identity and attribute verification	No direct transfer of sensitive health data Space, power, and computationally efficient	Poorly characterized and infrequently discussed in health data research	Explore practical applications with health data and characterize performance and privacy
Federated learning	Collaborative ML modeling while keeping training data local to data owners Decentralized or centralized for both data and model	Collaborative ML with theoretically any type of algorithm or data	Enables ML training with more diverse data Reduced computational load for institutions or devices Private data never moves beyond the firewalls of institutions or devices Provides a high level of data sovereignty to owners	Hard to establish a true baseline of privacy across learning system Scalability is dependent on collaboration and stable communication between otherwise sovereign and asymmetrical devices or institutions Aggregated data is not independent and identically distributed	Identify when a federated approach is the best choice for the specific reason of protecting data privacy Address challenges of interoperability Consistently characterize the tradeoffs between privacy, utility, and performance across different FL approaches to aid decision-making
Multi-party computation	Computation across multiple encrypted data sources while ensuring no party learns the private data of another Includes secret sharing, garbled circuits, oblivious transfer	Collaborative inference Third-party model training	Strong privacy protections for all participating parties No need for a trusted third-party High accuracy and precision	Communication and computational complexity are too high to use reasonably at scale and in resource-constrained environments Privacy-accuracy tradeoff	Develop more practical SMC solutions for resource-constrained environments and computations at scale
Synthetic Data	Synthesizing data to use instead of or in addition to real health data	Supports rapid development and benchmarking of ML algorithms Balance data that has uneven representation Augment datasets Measure utility loss of algorithmic PETs	It may be the most effective way to maximize privacy Increasingly easy and cost-efficient to implement	Limited methods to generate realistic data Limited types of data that can be synthesized Need to validate that synthetic data is representative of real data Should be restricted to secondary uses	Develop diverse methods to generate realistic synthetic data of all data types
Digital twinning	Virtual representations of what has been manufactured	A virtual counterpart to persons or hospitals to test tools like ML models	A real-time simulated environment without risk of exposing private data	Application in healthcare is primarily theoretical Privacy protections (e.g., risk of re-identification) are not well characterized	Develop practical applications of digital twins in healthcare Characterize privacy protections

*PET(s), privacy enhancing technology(ies); ML, machine learning; FL, federated learning; SMC, Secure multiparty computation*.

Although we were unable to draw clear “expert determinations” that could be delivered to decision-makers concerned about both privacy and computational or engineering performance, identified three promising examples of work that we each considered path-breaking in their treatment of PETs for health or health-relevant data (17, 22, 38). These articles carefully describe a PET, its applications, and the research performed to reach their conclusions, rendering both their methods and findings useful, of sufficient quality, and rigorously reported. Therefore, we strongly recommend that moving forward, multidisciplinary experts in privacy engineering and public health data begin by exploring the intricacies of these three studies, build on this work to bring relevance and internal validity to the fields of public health research and practice, and ultimately collaborate to provide formalized recommendations regarding the potential, utility, and acceptability of PETs to support public health research and practice.

## Author Contributions

SJ: conceptualization, project management, research, and composition. CF: research, composition, and design. RH-S: conceptualization, research, and composition. All authors contributed to the article and approved the submitted version.

## Conflict of Interest

RH-S reports unrelated contract work with the National Alliance Against Disparities in Patient Health. The remaining authors declare that the research was conducted in the absence of any commercial or financial relationships that could be construed as a potential conflict of interest.

## Publisher's Note

All claims expressed in this article are solely those of the authors and do not necessarily represent those of their affiliated organizations, or those of the publisher, the editors and the reviewers. Any product that may be evaluated in this article, or claim that may be made by its manufacturer, is not guaranteed or endorsed by the publisher.
